# Factors associated with adherence to antiretroviral therapy among HIV infected children in Kabale district, Uganda: a cross sectional study

**DOI:** 10.1186/s13104-018-3575-3

**Published:** 2018-07-13

**Authors:** Ignatius Wadunde, Doreen Tuhebwe, Michael Ediau, Gildo Okure, Arthur Mpimbaza, Rhoda K. Wanyenze

**Affiliations:** 1 Department of Health Policy, Planning and Management, Makerere University College of Health Sciences School of Public Health, P.O Box 7072, Kampala, Uganda; 2 Child Health and Development Centre, Makerere University College of Health Sciences School of Medicine, P.O. Box 6717, Kampala, Uganda; 3 Department of Disease Control and Environmental Health, Makerere University College of Health Sciences School of Public Health, P.O Box 7072, Kampala, Uganda

**Keywords:** Pediatric, Adherence, Antiretroviral therapy

## Abstract

**Objectives:**

This study was set out to assess the level of adherence to antiretroviral therapy (ART) and its determinants among children receiving HIV treatment in Kabale district, south western Uganda, in order to inform interventions for improving pediatric ART adherence.

**Results:**

Overall, 79% (121/153) of the children did not miss ART doses over the 7 days. Caregiver forgetfulness was the major reason for missing ART doses, 37% (13/35). Other reasons included transportation costs to the health facilities, 17%, (6/35) and children sitting for examinations in schools. Older children (11–14 years) were more likely to adhere to ART than the younger ones (0–10 years) (AOR = 6.41, 95% CI 1.31–31.42). Caregivers, who knew their HIV status, had their children more adherent to ART than the caregivers of unknown HIV status (AOR = 21.64: 95% CI 1.09–428.28). A significant proportion of children in two facilities 21.5% (32/153) missed ART doses within the previous week. Support for providers to identify clues or reminders to take drugs, extending HIV testing to caregivers and innovative models of ART delivery that alleviate transport costs to caregivers and allow sufficient drugs for children in school could enhance drug adherence among children.

## Introduction

Antiretroviral therapy (ART) improves health and prolongs the lives of persons with HIV [1–3] and in children, adherence to ART reduces viral load [4, 5], HIV/AIDS related morbidity [6] and mortality [7, 8]. Access to ART has rapidly expanded globally and in sub Saharan Africa especially with the most recent changes in the World Health Organization (WHO) guidelines to allow early treatment for HIV infected individuals [9]. However, implementation of ART among children 0–14 years, faces major challenges of adherence [10].

Better Socio-economic status and well tolerated regimens are associated with better adherence [11]. Other factors like socio-demographic and socio-cultural factors, side effects of ARVs, ART regimes, drug dosing [12, 13], duration on ART [14], health of child [5], child knowledge of their HIV status [15], and psychosocial factors such as stress, depression and anxiety [11] have also been associated with pediatric ART adherence [5, 16].

The Care giver report has been used as a simple and vital method in assessing pediatric ART adherence in Africa [5, 11, 17].

Thirteen percent of the people living with HIV/AIDS in Uganda are children [18, 19], and all HIV positive children less than 15 years (0–14) are initiated on ART irrespective of the CD4 count or WHO clinical staging [20]. To ensure retention in HIV care and adherence to HIV treatment, there should be constant supply of antiretroviral drugs (ARV’s), psychological support and HIV status disclosure by care giver with support of a counselor for children aged 10 and above [9, 20, 21].

In Kabale district, there were 564 HIV positive children aged 0–14 years and reports from the district health office indicated low adherence to ART. This study set out to determine the level of adherence and its associated factors among HIV infected children aged 0–14 years in Kabale district, so as to inform efforts for improving ART adherence among HIV infected children.

## Main text

### Method

We conducted a quantitative cross sectional study between June and August 2014 in Kabale district located in south western Uganda, with an estimated regional HIV prevalence of 5% [22, 23]. The district has twenty two health facilities providing pediatric ART; however, this study was conducted in two hospitals of Rugarama (private) and Kabale regional referral (public). These hospitals were deliberately selected because they provide HIV treatment to the largest number of HIV infected children in the district.

We interviewed 153 caregivers of HIV infected children aged 0–14 years receiving ART in the two hospitals. The caregivers had to be 18 years and above, age at which they could give informed consent and the eligible children were those who had been on ART for at least 3 months prior the interview. This study, since it was a cross sectional study, the sample size was determined from the formula for estimating sample sizes for prevalence studies [24].

The caregivers of the sampled children were interviewed using a pre-tested, semi-structured questionnaire translated into Rukiga, the predominant local language in Kabale district. The interviews were administered by trained study nurses fluent in Rukiga, the local language.

We assessed several factors that were suspected to influence ART adherence as informed from the literature review. The child related factors included age and health status of the child, knowledge of their HIV status and duration on ART [5, 15] [17]. The caregiver factors included the caregiver’s relationship with the child, stress and depression, age, sex, occupation, highest level of education attained, and duration as child’s caregiver [11, 15]. The drug regimen for each child was documented in addition to the other medication factors such as side effects of the ART, drug dosing and tolerability [5, 11, 15]. We also assessed caregiver forgetfulness to remind their HIV infected children on ART to take their medication on time, since it has been found to affect the child’s adherence [15].

The dependent variable was adherence to ART in the last 7 days as reported by the caregiver. Adherence measurement was based on the caregivers report of missed ART doses in the last 7 days prior to the interviews [15] and similar adherence studies used a 3 days recall [17]. It was characterized as “optimal adherence versus poor adherence”. Children whose caregivers reported no missed doses were considered to have optimal adherence to ART while those who reported one or more missed doses were considered to have poor adherence.

After questionnaires had been checked, the data was entered using Epi Info software and exported to Stata software for analysis. At Univariate analysis, categorical variables were analyzed using frequencies and proportions and continuous variables using means and standard deviations. The percentage of children with good adherence was calculated by dividing the number of care givers who reported that their children did not miss any dose within the last 7 days prior the interview by the total number of caregivers interviewed.

Bi-variable analysis was done to determine the relationship between each independent factor and adherence. Multivariate logistic regression was done on all factors that were significant after bi-variable analyses to identify factors independently associated with ART adherence. The association of independent variables with the dependent variable was measured using odds rations and the corresponding 95% confidence intervals (CI). A *p* value of < 0.05 was considered statistically significant.

### Results and discussion

All the 153 caregivers who were approached agreed to participate and were interviewed. Most of the sampled children (84.3%, 129/153) were enrolled from Kabale regional referral hospital. Most of the caregivers were in the 31–40 year age group, 40.1%, (62/153), and majority of the caregivers were females, 73.9% (113/153), had primary level of education 37.9% (58/153), were peasants, 47.6% (70/153) and 78.4% (120/153) were biological parents of the children. Of the 153 children, 56.2% (86/153) were females and the majority, 85.1%, (131/153) were above 5 years of age.

Details of the socio-demographics for the children and caregivers are shown in Table 1.

**Table 1:** Socio-demographic characteristics of caregivers and children on ART, Kabale, Uganda 2014

Characteristic	Frequency (n = 153)	Proportion (%)
Age of child		
0–4 years	23	14.94
5–10 years	66	42.86
11–14 years	65	42.21
Gender of child		
Female	86	56.21
Male	67	43.79
Age of care giver		
≤ 20 years	23	14.94
21–30 years	42	27.27
31–40 years	62	40.26
> 41 years	27	17.53
Sex of care giver		
Female	113	73.86
Male	40	26.14
Level of education of care giver		
None	23	15.03
Primary	58	37.91
Secondary	39	25.49
Tertiary	33	21.57
Occupation of care giver		
Peasant	70	47.62
Small business operator	37	25.17
Civil servant	25	17.01
Other^a^	15	9.8
Care giver relationship with child		
Biological parent	120	78.43
Sibling	5	3.27
Other relative	27	17.65
Not related	1	0.65

^a^Other (students, housewife, petty jobs)

#### Level of adherence to ART

Overall, 79.1% (121/153) of the children did not miss any ART doses over the 7 days. Thirty-five children (20.9) missed at least one dose within a period of 7 days. The commonest reasons for missing doses were forgetfulness, 34% (13/35), transportation costs to the health facilities, 17% (6/35) and children sitting for examinations at school, 17% (6/35).

Seventeen caregivers reported various side effects of ART including dizziness, 23% (4/17), vomiting, 18% (3/17), stomach pain, 11% (2/17), rashes, 18% (3/17), headaches, 18% (3/17) and fever, 11% (2/17) (Table 2).

**Table 2:** Reasons for missing ART doses and side effects of ART among HIV children on ART, Kabale, Uganda 2014

Characteristic	Frequency	Proportion (%)
Reasons for missing dose	(n = 35)	
Caregiver forgetfulness	13	37.1
Transportation problems	6	17.1
School examinations	6	17.1
Children went playing	5	14.3
Child vomited drug	4	11.4
Drug run out	1	2.9

#### Factors associated with adherence to antiretroviral therapy

After controlling for child age, duration on ART, knowledge of their HIV status, age of caregiver, caregiver level of education, caregiver relationship with child and caregiver knowing their HIV status, child age and caregiver knowledge of their HIV status had significant associations with adherence.

Older children (11–14 years) were more likely to adhere to ART than the younger ones (0–10 years) AOR 6.41 (95%CI 1.31–31.42) p-value 0.022. Children of Caregivers who knew their HIV status were more likely to adhere to ART than those whose caregivers did not know their HIV status AOR 21.64 (1.09–429.24) p-value 0.044 (Table 3).

**Table 3:** Factors associated with adherence to ART doses among HIV infected Children 0–14 years in Kabale district, Uganda

Variable	Adherent							p-value
	Yes	No	COR	95% CI		AOR	95% CI	
	(n = 121)	(n = 32)						
Study site								
Rugarama hospital	3 (9.38)	20 (16.95)	1.0					
Kabale hospital	29 (90.63)	98 (83.05)	0.5	(0.14–1.83)				
Age of child								
0–10 years	19 (59.38)	68 (57.14)	1.0			1.0		
11–14 years	13 (40.63)	51 (42.86)	1.1	(0.49–2.42)		6.4	(1.31–31.42)	0.022*
Gender of child								
Female	16 (50.00)	68 (58.12)	1.0					
Male	16 (50.00)	49 (41.88)	0.7	(0.33–1.58)				
Child duration on ART								
0–4 years	14 (43.75)	58 (49.15)	1.0			1.0		
5 years above	18 (56.25)	60 (50.85)	0.8	0.37–1.77		1.2	(0.39–3.58)	0.768
Childs’ health								
Not sick by time of interview	28 (87.50)	108 (91.5)	1.0					
Sick time of interview	4 (12.50)	10 (8.47)	0.7	(0.19-2.22)				
Sex of care giver								
Female	23 (71.88)	88 (74.58)	1.0					
Male	9 (28.13)	30 (25.42)	0.9	(0.36–2.09)				
Age of care giver								
≤ 30 years	13 (40.63)	51 (42.86)	1.0			1.0		
30 years above	19 (59.38)	68 (57.14)	0.9	(0.41–2.01)		0.8	(0.44–1.49)	0.499
Level of education of care giver								
None	4 (12.50)	19 (16.10)	1.0					
Primary	14 (43.75)	43 (36.44)	0.7	(0.19–2.22)		0.38	(0.06–2.40)	0.307
Secondary	4 (12.50)	33(27.97)	1.7	(0.39–7.76)		2.26	(0.2–20.95)	0.474
Tertiary	10 (31.25)	23 (19.49)	0.5	(0.13–1.79)		0.16	(0.02–1.21)	0.077
Occupation of care giver								
Formal employment	23 (76.67)	96 (84.21)	1.0					
Informal employment	7 (23.33)	18 (15.79)	0.6	(0.23–1.66)				
Care giver relationship with child								
Biological parent	24 (77.42)	93 (78.81)	1.0			1.0		
Other relative	7 (22.58)	25 (21.19)	0.9	(0.36–2.39)		0.3	(0.07–1.70)	0.189
Duration as caregiver of child								
0–4 years	13 (40.63)	51 (42.86)	1.0					
5 years above	19 (59.38)	68 (57.14)	0.9	(0.41–2.02)				
Care giver’s health								
Not sick a week before interview	25 (80.65)	100 (85.5)	1.0					
Sick a week before interview	6 (19.35)	17 (14.5)	0.7	(0.25–1.99)				
Caregiver knowledge of his/her HIV status								
Do not know their HIV status	5 (16.13)	9 (7.63)						
Know their HIV status	26 (83.87)	109 (92.4)	2.3	0.71–7.63	0.15	21.6	1.09–428.2	0.044*
Care giver stress								
Not stressed	29 (90.63)	97 (81.5)	1.0					
Stressed	3 (9.38)	22 (18.5)	2.2	(0.61–7.94)				
Drug tolerability								
Child does not find a problem swallowing	31 (96.88)	110 (97.4)	1.0					
Child finds swallowing the drug a problem	1 (3.13)	3 (2.65)	0.9	(0.08–8.48)	0.886			
Drug dosage complexity								
Child find dose easy to take	30 (93.75)	105 (90.5)						
Child find dose not easy to take	2 (6.25)	11 (9.48)	1.6	(0.33–7.53)	0.569			
Side effects of the ARV’s								
Child has never experienced drug side effects	26 (83.87)	107 (89.9)						
Child has experienced a side effect due to the ARV’s	5 (16.13)	12 (10.1)	0.6	(0.19–1.81)	0.346			

*Statistically significant

Caregiver forgetfulness was a major (37%) reason for missing ART doses. This can be improved by advising the caregivers to give the children the medicines consistently at the same convenient time of the day and using clues to remind them to give the child their drugs [25, 26]. The other reason for missing ART doses were transportation to facilities for drug refills. Transportation cost as a limitation for appointment keeping and drug refills has been reported by studies among children and adults [27, 28]. Community based refills for stable patients can alleviate such challenges and also improve efficiencies for service delivery [29].

Our study found that older children (11 years and above) were more likely to adhere to ART than younger ones (0–10 years), and this is in line with findings from Ethiopia [30]. Older children have better awareness and appreciation of the negative effects of poor ART adherence, especially if their HIV status has been disclosed to them [25]. Providers should thus pay more attention to the younger children and provide support to caregivers to bridge the gaps.

Our study also found that caregivers who knew their HIV status, had their HIV infected children more likely to adhere to ART compared to the children of the caregivers who did not know their HIV status. This implies that all caregivers of HIV infected children should be advised to know their HIV status, enhances their HIV infected children to adhere to their medication.

### Conclusions

The level of adherence to antiretroviral therapy was found to be sub optimal, a significant proportion of children, 21% (35/153) missed their drugs.

Caregiver knowledge of their HIV status was associated with pediatric ART adherence, so there is need to integrate efforts to enhance caregivers of HIV infected children to know their HIV status. Caregiver forgetfulness and transportation challenges also led to missed doses.

## Limitations

In our study, we recognize a major limitation of the use of a small sample size (153) that gave rise to very wide confidence intervals. Caregiver reports of missed ART doses to assess adherence, is also a less objective measure of adherence because it leads to over estimation of adherence, recall bias and social desirability bias.

## Abbreviations

ART: adherence to antiretroviral therapy
ARV’s: antiretroviral drugs
CI: confidence interval
HDREC: Higher Degrees Research and Ethics Committee
WHO: World Health Organization
